# Vasodilator-stimulated phosphoprotein-guided Clopidogrel maintenance therapy reduces cardiovascular events in atrial fibrillation patients requiring anticoagulation therapy and scheduled for percutaneous coronary intervention: a prospective cohort study

**DOI:** 10.1186/s12872-018-0853-x

**Published:** 2018-06-18

**Authors:** Chaoyue Hu, Xumin Zhang, Yonghua Liu, Yang Gao, Xiaohong Zhao, Hua Zhou, Yu Luo, Yaling Liu, Xiaodong Wang

**Affiliations:** 1 Key Laboratory of Arrhythmias of the Ministry of Education of China, Tongji University School of Medicine, Shanghai, 200092 China; 2 Department of Cardiology, Shanghai East Hospital, Tongji University School of Medicine, 150 Jimo Road, Shanghai, 200120 China; 3 Cardiovascular Medicine of Baoshan People’s Hospital of Yunnan Province, Baoshan, 678000 China; 4 Department of Anesthesiology, Renji Hospital, Shanghai Jiaotong University School of Medicine, 160 Pujian Road, Shanghai, 200127 China

**Keywords:** Atrial fibrillation, Anticoagulation, Vasodilator-stimulated phosphoprotein, Clopidogrel, Percutaneous coronary intervention

## Abstract

**Background:**

In a previous study, we found that titrating clopidogrel maintenance doses (MDs) according to vasodilator-stimulated phosphoprotein (VASP) monitoring minimised the rate of major adverse cardiovascular and cerebral events (MACCE) after percutaneous coronary intervention (PCI) without increasing bleeding in patients with high on-treatment platelet reaction to clopidogrel. This study aimed to investigate whether VASP-guided clopidogrel MD could reduce thromboembolism and bleeding in atrial fibrillation (AF) patients requiring anticoagulation and scheduled for PCI.

**Methods:**

AF patients scheduled for PCI were recruited between July 2014 and July 2016. These patients were allocated into VASP-guided (*n* = 250) and control (*n* = 253) groups depending on the clopidogrel MD profile. In the VASP-guided group, clopidogrel MD was titrated by the platelet reactivity index (PRI), whereas in the control group, clopidogrel MD was fixed at 75 mg per day. The primary endpoint was MACCE and secondary endpoints were thrombolysis in myocardial infarction (TIMI) major and minor bleeding 1 year after PCI.

**Results:**

Five hundred and three patients were included in the present study, with 1-year data available for 95.6% patients. The average CHA_2_DS_2_-VASc score of the whole population was 3.7 ± 0.7 and the average HAS-BLED score was 3.2 ± 0.4. MACCE was less in the VASP-guided group than in the control group (2.5% vs. 5.0%, *P* = 0.02). The incidence of major bleeding was comparable between both groups (3.0% vs. 2.8%, *P* = 0.72) and minor bleeding was higher in the VASP-guided group than in the control group (15.3% vs. 9.7%, *P* = 0.03). Kaplan-Meier analysis indicated that there was no difference in survival between both groups (log-rank test, *P* = 0.68).

**Conclusions:**

In AF patients requiring anticoagulation and scheduled for PCI, VASP-guided antiplatelet therapy reduced major cardiovascular and cerebral adverse events, accompanied by increased minor bleeding events.

**Trial registration:**

The present study was retrospectively registered in the Chinese Clinical Trial Registry, A Primary Registry of the International Clinical Trial Registry Platform, World Health Organisation (Registration no: ChiCTR-IOR-17013854). The registered date was December 11, 2117.

## Background

Atrial fibrillation (AF) is the most common abnormal cardiac rhythm, having a high risk of thromboembolism and causing detrimental clinical outcomes. About 5 to 10% of AF patients requiring oral anticoagulation undergo coronary stent implantation [1]. This spectrum of patients requires combined therapy with oral anticoagulation, aspirin, and P2Y12 receptor blocker, which is known as triple therapy (TT). The most common combination currently consists of the vitamin K antagonist warfarin, and aspirin and clopidogrel.

Although TT has a potentially beneficial antithrombotic effect, prolonged TT therapy may increase bleeding risk. Current guidelines recommend the concomitant administration of TT for short consecutive periods [2]. Clopidogrel, preferred to other novel P2Y12 receptor blockers (prasugrel and ticagrelor), is the only thienopyridine recommended in guidelines. In our previous study, we found that titrating clopidogrel maintenance doses (MDs) according to vasodilator-stimulated phosphoprotein (VASP) monitoring minimised the rate of major adverse cardiovascular and cerebral events (MACCE) after percutaneous coronary intervention (PCI) without increasing bleeding in patients with high on-treatment platelet reaction (HTPR) to clopidogrel at 1-year follow-up [3].

## Methods

### Aim

In the present study, we aimed to clarify whether VASP-guided clopidogrel MD could decrease adverse clinical events in AF patients requiring anticoagulation and scheduled for PCI.

### Study design and patients

This was a prospective cohort study that included consecutive patients with stable coronary artery disease who had AF requiring anticoagulation and were scheduled for PCI. Patients were included if they 1) were older than 18 years and no more than 80 years, 2) had a preexisting diagnosis of paroxysmal, persistent, or permanent AF with anticoagulation therapy warfarin, 3) had effort angina pectoris despite optimal medical therapy, and 4) had silent ischaemia on radionuclide imaging. Exclusion criteria included cardiac arrest, New York Heart Association III/IV function, platelet count < 100 × 10^9^/L, creatinine clearance rate < 25 mL/min.

We included patients from Shanghai East Hospital, a teaching hospital of Tongji University serving a population of approximately 5,500,000, with 12 intervention specialists and 2000 PCI procedures each year. Five hundred patients were randomly allocated to the VASP-guided and control groups. A statistician performed the allocation. Blinding was used for the participants and/or researchers. We collected data on demographic and clinical characteristics, prothrombotic risk factors, and antithrombotic therapy strategies before and after PCI. Patients’ thromboembolism risk was evaluated by the CHA_2_DS_2_-VASc (Congestive heart failure, 1 point; Hypertension, 1 point; Age ≥ 75 years, 2; Diabetes mellitus, 1 point; Stroke/transient ischaemic attack, 2; Vascular disease, 1 point; Age 65–74 years, 1 point; Sex category for 1) score. Bleeding risk was assessed by the HAS-BLED (Hypertension, 1; Abnormal renal and liver function, 1 point each; Stroke/thromboembolism, 1 point; Bleeding history, 1 point; Labile INR [international normalised ratio], 1 point; Elderly [age > 65 years], 1 point; Drug consumption and alcohol abuse, 1 point each) score. All procedures were performed according to the rules of the Ethical Committee on human clinical trials and according to the Helsinki Declaration revised in 2008. Informed written consent was obtained from all participants. The name of the registry was “Prospective randomized controlled study of anti-platelet therapy in atrial fibrillation patients undergoing percutaneous coronary intervention”.

### Definitions

AF was defined as paroxysmal, persistent, permanent, or unknown according to guidelines [4]. Stroke was defined as the sudden loss of neurologic function, which was classified into ischaemic or haemorrhagic and verified by brain computed tomography or magnetic resonance imaging [5]. Systemic embolism was diagnosed as acute vascular obstruction of the limbs or any organ and was verified by angiography. Acute myocardial infarction (AMI) was defined according to the universal definition of the ESC/ACCF/AHA/WHF [6]. Stent thrombosis (ST) was defined according to the Academic Research Consortium standard [7]. Thrombolysis in myocardial infarction (TIMI) major bleeding included intracranial or clinically significant haemorrhage with a haemoglobin decrease > 50 g/L according to the TIMI criteria [8]. Minor bleeding was also defined according to the TIMI criteria [8].

### PCI

PCI was performed in accordance with international guidelines, using a standard technique, through the radial or femoral route [9]. A drug-eluting stent (DES) was used based on the angiography outcome. An intravenous bolus of unfractionated heparin (100 IU/kg) was administered immediately before the procedure. The administration of glycoprotein IIb/IIIa inhibitors was decided on by the attending cardiologists.

### Antithrombotic therapy and clopidogrel modification

Warfarin was stopped 3 days before PCI and patients were treated with low-molecular-weight heparin until 12 h before PCI. A combined loading dose of 300 mg aspirin and 600 mg clopidogrel was used before PCI. After PCI, a combined administration of 100 mg aspirin, 75 mg clopidogrel, and INR-monitored warfarin was continued for 3 months according to guidelines [9]. The clopidogrel MD (Plevix, 75 mg per tablet, SANOFI, France, and clopidogrel bisulfate tablets, 25 mg per tablet, SALUBRIS, China) fluctuated between 75 and 225 mg for at least 1 year. All patients were treated with TT for 3 months, followed by clopidogrel plus warfarin for 9 months. VASP-adjusted clopidogrel MD commenced at the beginning of 3 months and was modified according to the VASP index in order to keep the platelet reaction index (PRI) below 50%. VASP was monitored at 3, 6, 9, and 12 months after PCI. The stepwise increase of clopidogrel dosage was adopted. After the first PRI monitoring, 100 mg clopidogrel was administered if PRI > 50%. The second PRI monitoring was undertaken 6 months after PCI, and 125 mg clopidogrel was administered if PRI was still > 50%. Subsequent additional doses were similar to the previous doses. Another 25 mg clopidogrel was given if PRI > 50% after every 3 months. The maximum clopidogrel MD at the end of 1 year was 175 mg. If PRI < 25%, clopidogrel MD was decreased to 75 mg. If PRI > 25% and was < 50%, the dose was not changed and the determined dose was maintained.

### Blood samples

Three months after PCI, blood samples were drawn from the antecubital vein once every 3 months. The initial blood was removed to avoid platelet activation induced by the puncture action. Blood was transferred into a tube with 3.8% trisodium citrate. The tube was gently inverted up and down 3 to 5 times and was immediately sent to the monitoring laboratory.

### VASP phosphorylation test

The VASP phosphorylation test was performed more than 1 h after blood collection by an experienced technician using Platelet VASP kits (Becton Dickinson, USA) according to the instruction manual [10]. Briefly, blood samples were mixed in vitro with adenosine diphosphate (ADP) and/or prostaglandin E1 (PGE1). Each blood sample was incubated with a 16C2FITC antibody, followed by a goat anti-mouse fluorescence staining in isothiocyanate polyclonal reagent. Flow cytometric monitoring was conducted with a Coulter EPICS XL cytometer (CA, U.S.A). The platelet group was identified on its side scatter and forward distributions. Every 3000 platelet events were gated and analysed for mean fluorescence intensity (MFI) using flow cytometry. The MFI corresponding to each experimental situation (ADP and ADP + PGE1) was determined to calculate a ratio directly related to the VASP phosphorylation value. The ratio, [(MFI_PGE1_-MFI_ADP + PGE1_)/MFI_PGE1_] × 100%, which indicates a percentage of platelet reactivity, is expressed as a PRI corresponding to a ratio of activated platelets versus resting platelets.

### INR monitoring

For all patients who received warfarin, the predicted INR was set between 2.0 and 2.5 according to guidelines [2]. INR was regularly monitored after discharge. In the beginning, INR evaluations were performed every week after discharge. If the INR achieved the target range after monitoring three consecutive times, the measurement was then taken monthly.

### Clinical endpoint

For the clinical follow-up, the primary endpoint was designated as the occurrence of MACCE, involving cardiovascular death, myocardial infarction (MI), target vessel revascularisation (TVR), ST, systemic embolism, and stroke. Secondary endpoints were defined as TIMI major and minor bleeding.

### Follow-up

The clinical follow-up involved a clinic visit or telephone interview at 3, 6, 9, and 12 months after PCI. If a dose adjustment was made at 9 months, the patients were followed up for another 1 year from then on. The MD modification was as before. All patients with symptoms during the visit and interview were evaluated by at least two cardiologists. Death or MI events were recorded by hospital admission staff or community service.

### Statistics

Continuous variables are described as mean ± SD and categorical variables as numbers and percentages. Differences between treatment groups were tested with one-way or two-way repeated-measures ANOVA, followed by Bonferroni correction for intergroup comparisons. Comparison between categorical variables was performed with the Chi-square test or Fisher exact test if frequencies were no more than 5. Event-free survival rates in different groups were calculated by Kaplan-Meier survival analysis and compared by the log-rank test. A two-sided *P*-value of < 0.05 was considered statistically significant. Data statistics were performed using Prism 5.0 (GraphPad Software, CA, USA).

## Results

**Fig. 1:**
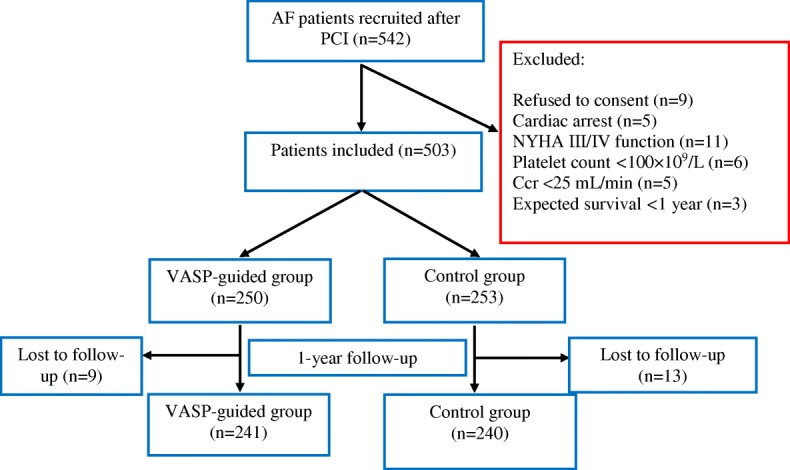
Study flowchart and follow-up. AF: atrial fibrillation; CCr: creatinine clearance rate; NYHA: New York Heart Association; PCI: percutaneous coronary intervention; VASP: vasodilator-stimulated phosphoprotein

We prospectively recruited 503 patients with AF requiring anticoagulation and who underwent PCI between July 2014 and July 2017; 481 patients (VASP-guided, *n* = 241; control, *n* = 240) completed the 1-year follow-up (Fig. 1). In the VASP-guided and control groups, 89.7 and 89.9% of patients, respectively, had paroxysmal or persistent/permanent AF. The clinical and procedural characteristics and other medical histories were comparable between both groups. In the VASP-guided and control groups, the CHA_2_DS_2_-VASc score was 3.7 ± 0.6 and 3.8 ± 0.9, respectively, whereas, the HAS-BLED score was 3.1 ± 0.4 and 3.3 ± 0.5, respectively, indicating high thrombotic and bleeding risk profiles in both groups. The average INR on the day of the procedure was 1.2 ± 0.6 and 1.3 ± 0.7, respectively, indicating unsatisfactory warfarin therapy in both groups (Table 1).

**Table 1:** Clinical, medical, and procedural characteristics

Characteristics	VASP-guided group	Control group	*P*-value
	(*n* = 241)	(*n* = 240)	
Age (y)	64.4 ± 3.2	62.8 ± 4.3	0.58
Age ≥ 75 years	61(25.3)	64(26.7)	0.72
Men	128(53.4)	122(50.8)	0.62
BMI (kg/m^2^)	26.7 ± 4.2	25.3 ± 2.1	0.88
Current Smoker	87(36.2)	96(40.1)	0.55
AF category			
Paroxysmal	92(38.4)	96(40.2)	0.7
Persistent/permanent	123(51.3)	119(49.7)	0.84
Unknown	26(10.3)	25(10.1)	0.98
Medical history			
Diabetes	68(28.4)	75(31.3)	0.43
Hypertension	141(58.8)	144(60.3)	0.57
Previous heart failure	41(17.1)	52(21.9)	0.19
Previous stroke or	25(10.3)	28(11.6)	0.65
thromboembolism			
Previous bleeding	16(6.7)	13(5.5)	0.53
CHA2DS2 -VASc Score	3.7 ± 0.6	3.8 ± 0.9	0.87
CHA2DS2 -VASc Score ≥ 2	174(72.4)	168(70.3)	0.35
HAS-BLED Score	3.1 ± 0.4	3.3 ± 0.5	0.76
HAS-BLED Score ≥ 3	61(25.3)	59(24.8)	0.64
LVEF	52.3 ± 4.4	51.0 ± 3.9	0.59
Treatment on admission			
Previous aspirin	31(12.8)	33(14.0)	0.21
Previous clopidogrel	13(5.4)	14(7.3)	0.48
Previous oral anticoagulation	131(54.3)	119(49.7)	0.65
Indication for the catheterisation procedure			
Stable angina	188(78.5)	168(70.2)	0.74
Silent myocardial ischaemia	52(21.5)	71(29.8)	0.17
Platelet (×109/L)	202 ± 23	183 ± 17	0.28
Cr (μmmol/L)	98.3	89.4	0.13
Mean INR on day of procedure	1.2 ± 0.6	1.3 ± 0.7	0.72
Pharmacotherapy			
RAS inhibitors	212(88.2)	200(83.5)	0.61
Statins	237(98.3)	237(98.7)	0.98
β-Blockers	123(51.4)	132(55.3)	0.76
Digoxin	32(13.4)	41(17.2)	0.25
Amiodarone	62(25.9)	46(19.4)	0.09
Procedural characteristics			
No. of lesions treated per patient	1.4	1.5	0.88
No. of stents per patient	1.3	1.2	0.64
Patients receiving DES	241(100)	240(100)	1
GP IIb/IIIa inhibitors	61(25.4)	67(28.1)	0.38

Values are presented as mean ± SD or n (%)

*AF* atrial fibrillation, *BMI* body mass index, *Cr* creatinine, *GP* glycoprotein, *INR* international normalised ratio, *LVEF* left ventricular ejection fraction, *RAS* renin-angiotensin system

### Antithrombotic mediation after discharge

For patients prescribed antithrombotic therapy after discharge, there was high compliance in both groups. The median duration of TT and clopidogrel plus warfarin was 3.2 and 9.5 months, respectively. There was no difference in duration between CHA_2_DS_2_-VASc 1 or ≥ 2 and HASBLED ≤2 or ≥ 3 (Table 2).

**Table 2:** Antithrombotic drug regimen at discharge

Medication	Median duration (months)				
	CHA_2_DS_2_-VASc = 1	CHA_2_DS_2_-VASc≥2	HASBLED≤2	HASBLED ≥3	*P*-value
Aspirin	2.3 ± 0.7	3.2 ± 1.1	3.8 ± 0.9	3.3 ± 0.9	>0.05
Clopidogrel	11.2 ± 2.8	12.9 ± 1.4	12.6 ± 2.5	10.3 ± 1.6	>0.05
Warfarin	10.5 ± 2.8	12.4 ± 1.3	11.8 ± 2.1	10.8 ± 2.7	>0.05

### PRI

We analysed PRI at a mean time of 3, 6, 9, and 12 months after the PCI procedure. The baseline PRI showed no significant difference between both groups (73.5 ± 12.7% [VASP] vs 68.4 ± 17.2% [control], *P* = 0.4). PRI in the VASP-guided group decreased significantly (73.5 ± 12.7%, 32.3 ± 4.9%, 35.5 ± 6.7%, and 29.8 ± 7.3% at 3, 6, 9, and 12 months after randomisation, respectively; *P* = 0.001); PRI in the control group also decreased, but not significantly (68.4 ± 17.2%, 48.5 ± 13.2%, 51.6 ± 19.8%, and 65.3 ± 17.2%, respectively; *P* > 0.5). Compared to the control group, PRI in the VASP-guided group was significantly lower (*P* = 0.04, 0.03, and < 0.001 at 6, 9, and 12 months after randomisation, respectively) (Table 3).

**Table 3:** Platelet Reactivity Index (PRI) in the two groups during the 1-year study

	PRI (mean ± SD, %)				
	3 months	6 months	9 months	12 months	*P*-value*
	after randomisation				
Control group	68.4 ± 17.2	48.5 ± 13.2	51.6 ± 19.8	65.3 ± 17.2	>0.05
VASP-guided group	73.5 ± 12.7	32.3 ± 4.9	35.5 ± 6.7	29.8 ± 7.3	0.001
*P*-value#	0.4	0.04	<0.001	0.03	<0.001

*VASP* vasodilator-stimulated phosphoprotein

*comparison between 3, 6, 9, and 12 months

^#^comparison between the control group and VASP-guided group

### Clopidogrel dose modification

**Fig. 2:**
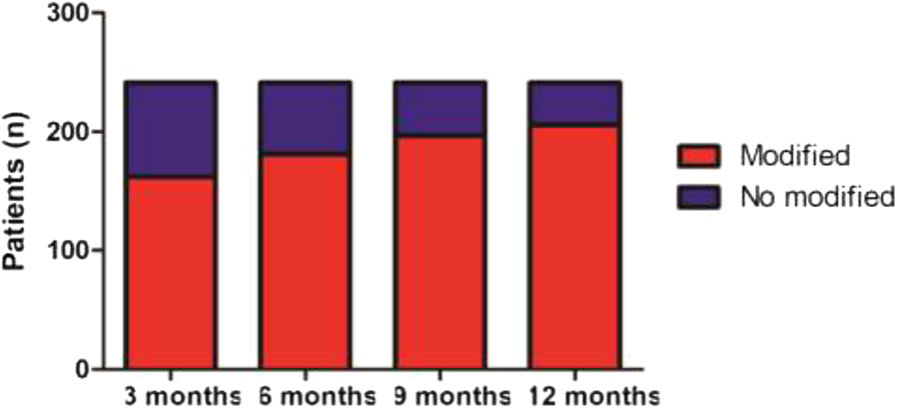
Patient distribution according to the modified or unmodified clopidogrel maintenance dose in the VASP-guided group. VASP: vasodilator-stimulated phosphoprotein

**Fig. 3:**
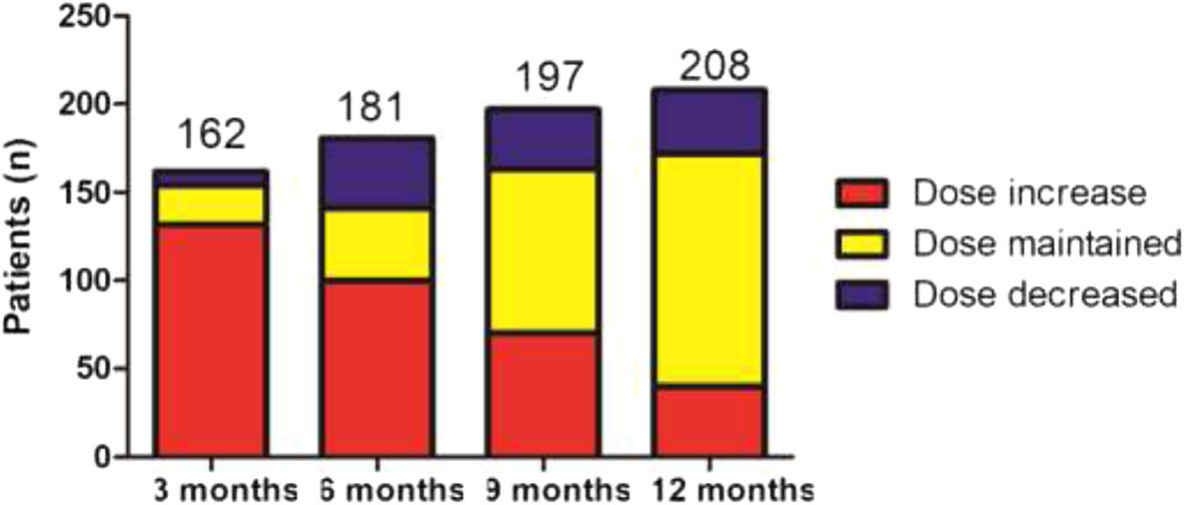
Patient distribution according to the clopidogrel maintenance dose modification profile in the VASP-guided group. VASP: vasodilator-stimulated phosphoprotein

Clopidogrel MD in the VASP-guided group was modified according to PRI. The number of patients that required clopidogrel MD individualisation was 162 (67.3%), 181 (75.4%), 197 (81.9%), and 208 (86.3%) at 3, 6, 9, and 12 months, respectively (Fig. 2). Regarding MD according to PRI at 3, 6, 9, and 12 months, 132 (81.5%), 100 (55.2%), 70 (35.5%), and 40 (19.4%) patients, respectively, had increased MD, 22 (13.6%), 41 (22.6%), 93 (47.2%), and 130 (63.1%) patients, respectively, had unchanged MD, while 8 (4.9%), 40 (22.1%), 34 (17.3%), and 36 (17.5%) patients, respectively had decreased MD (Fig. 3). At the study’s completion, 33 of 241 (13.7%) patients in the VASP-guided group still had HTPR> 50% (data not shown).

### INR monitoring

During the 1-year follow-up, INR was measured at least every month. The representative value at 1, 3, 6, 9 and 12 months are listed in Table 4. INR increased at 12 months compared to baseline only in patients with CHA_2_DS_2_-VASc score ≥ 2 (from 1.9 ± 0.3 to 2.5 ± 0.8, *P* < 0.05).

**Table 4:** INR monitoring during the 1-year follow-up

Categories	1 mo	3 mo	6 mo	9 mo	12 mo	*P*-value
VASP-guided group	1.8 ± 0.3	2.1 ± 0.5	1.9 ± 0.7	2.0 ± 0.2	2.2 ± 0.4	0.33
Control group	1.7 ± 0.9	2.0 ± 0.3	2.1 ± 0.5	1.9 ± 0.6	2.1 ± 0.2	0.48
CHA2DS2-VASc Score = 1	2.0 ± 0.2	1.7 ± 0.4	2.2 ± 0.9	2.0 ± 0.8	2.1 ± 0.7	0.56
CHA2DS2-VASc Score ≥ 2	1.9 ± 0.3	1.6 ± 0.6	2.3 ± 0.5	2.6 ± 0.9	2.5 ± 0.8	< 0.05
HAS-BLED Score < 2	1.6 ± 0.5	1.8 ± 0.4	2.0 ± 0.6	2.1 ± 0.4	2.2 ± 0.7	0.14
HAS-BLED Score ≥ 3	1.7 ± 0.2	1.9 ± 0.5	2.1 ± 0.6	2.2 ± 0.4	2.1 ± 0.8	0.35

P-value: 1 month vs. 12 months

### Clinical outcomes during follow-up

**Fig. 4:**
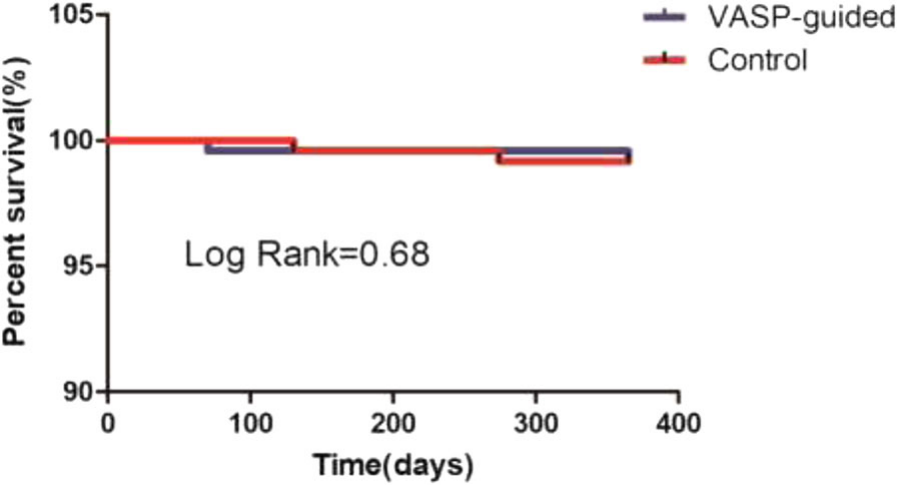
Kaplan-Meier curves of survival during the 1-year follow-up. VASP: vasodilator-stimulated phosphoprotein

Complete follow-up (median 365 days; range 300 to 395 days) was recorded in 95.6% of the whole cohort. The clinical events recorded in the two groups are shown in Table 5. The incidences of TVR and all MACCE were higher in the control group than in the VASP-guided group, whereas the occurrence of cardiovascular death, MI, ST, systemic embolism, and stroke was not significantly different between both groups. Regarding the secondary endpoints, major bleeding incidence was comparable between both groups, whereas the rate of minor bleeding was significantly higher in the VASP-guided group (15.3% vs 9.7%, *P* = 0.032). Kaplan-Meier survival analysis demonstrated that there was no statistical difference between the VASP-guided and control groups during the 1-year follow-up (log-rank test *P* = 0.68) (Fig. 4).

**Table 5:** Outcomes during the follow-up

Outcomes	VASP-guided group	Control group	*P*-value
	*n* = 241	*n* = 240	
Cardiovascular death	1(0.4)	2(0.8)	0.34
MI	1(0.4)	2 (0.8)	0.34
TVR	1(0.4)	3(1.3)	0.03
Stent thrombosis	1(0.4)	1(0.4)	0.21
Systemic embolism	1(0.4)	2(0.8)	0.34
Stroke	1(0.4)	2(0.8)	0.34
All MACCE	6(2.5)	12(5.0)	0.02
TIMI major bleeding	7(3.0)	6(2.8)	0.72
TIMI minor bleeding	37(15.3)	23(9.7)	0.03

Values are presented as n(%)

*MACCE* major adverse cardiovascular and cerebral event, *MI* myocardial infarction, *TIMI* thrombolysis in myocardial infarction, *TVR* target vessel revascularisation

## Discussion

To our knowledge, this is the first prospective study to show that individualised clopidogrel MD according to platelet function reduced the incidence of MACCE in AF patients requiring anticoagulation and scheduled for PCI. However, an increase in minor bleeding was noted. The study shows that our patients had a high risk of stroke and bleeding. Our clinical data demonstrate the protective effect of individualised clopidogrel MD in patients with AF undergoing PCI by decreasing the incidence of adverse clinical events, without increasing major bleeding.

Owing to lack of well-founded evidence to date, there has been no consensus on the optimal therapy regarding the antithrombotic strategy for AF patients requiring chronic anticoagulation and coronary stent implantation. Most previous studies evaluating TT have either been small-scale retrospective or case-control clinical trials focusing on bleeding risk. Thus, there is a lack of evidence to support optimal medical therapy regarding the cardiovascular efficacy of different antithrombotic regimens. In the largest observational study of AF patients with stable coronary artery disease in Denmark, the addition of antiplatelet therapy (either aspirin or clopidogrel) to vitamin K antagonist therapy decreased recurrent cardiovascular events or thromboembolism but increased bleeding significantly [11]. In that study and in the present study, the high CHA_2_DS_2_-VASc score indicated a high thrombotic risk in both cohorts. The greater number of bleeding events in the previous study might be attributed to racial differences or the fixed TT strategy.

In the Karjalainen et al. [12] case-control study, warfarin plus aspirin failed to prevent more cardiovascular events. However, this combination increased the risk for stent thrombosis. In the study by Ruiz-Nodar et al. [13] regarding combined therapy with coumarins, aspirin, and clopidogrel, the incidence of adverse events in TT was low, with no increase in minor and major bleeding compared to dual antiplatelet therapy (DAPT). The prospective multicentre registry study, STENTICO, demonstrated an increase in severe and moderate GUSTO bleeding in TT compared to DAPT [14]. In addition, the AVIATOR Registry study [15], involving patients that received TT or DAPT, showed similar MACE rates, with a higher BARC ≥2 bleeding when discharged. In a prospective multicentre study [16], TT was compared to DAPT in patients with AF undergoing PCI. The results showed that patients with a low CHA_2_DS_2_-VASc score had a high risk of bleeding without any benefit in reducing thromboembolic events. It also demonstrated that TT decreased the thromboembolism rate at the expense of an increase in major bleeding in patients with high CHA_2_DS_2_-VASc scores. These studies show the variability of antithrombotic agents in this kind of patients. There is no one-size-fits-all strategy for balancing thrombotic and bleeding risk.

In the present study, TT was used for an average of 3 months in all patients, which might be one reason major bleeding risk did not increase during the 1-year follow-up. Recently, the ISAR-TRIPLE trial [17] evaluated the effect of clopidogrel in addition to concomitant aspirin and warfarin following DES implantation. The study showed no significant difference in MACCE between 6 months and 6 weeks of TT. Furthermore, a longer duration of TT did not increase the bleeding risk in this study. This added evidence supports limiting the duration of DAPT and individualising therapy based on the patient’s risk profile. In a recent study [18], 568 patients receiving TT were prospectively investigated according to a 1-month or > 1-month regimen. The endpoints of primary safety and secondary bleeding were not significantly different between both groups. The study suggested that 30-day TT had similar clinical outcomes compared to longer TT durations. Because of the involvement of high-thrombotic-risk patients in the present study and guideline recommendations [2], we administered TT for 3 months.

Few trials have focused on antithrombotic therapy in Asian patients, particularly Chinese patients, who have undergone PCI and require oral anticoagulation. A prospective study involving 142 Chinese patients discharged with TT demonstrated a notable decrease in stroke and MACCE and its major bleeding risk might have fallen within acceptable ranges if the INR was strictly monitored [19]. In that study, the overall major adverse events in the TT group was 8.8%, and major and minor bleeding were 2.9 and 8.8%, respectively. In our present study, the major adverse events were 2.5% in the VASP-guided group and 5.0% in the control group, and major and minor bleeding were 2.8 to 3% and 9.7 to 15.3%, respectively. The difference between our study and theirs might be explained by the thrombotic risk of patients; the present study had a high-thrombotic-risk cohort (CHA_2_DS_2_-VAScScore ≥2, 71.5%). Another study with a small cohort of 37 patients requiring TT was conducted in South Korea [20]. The authors concluded that warfarin therapy reduced major adverse events without increasing bleeding risk. In that study, the percentage of CHADS_2_ score ≥ 2 was 56.8%, whereas, all major adverse events and any bleeding were 0.97 and 1.94%, respectively. These differences may be attributed to the nationality and sample size, although this study was conducted in Asia.

Inter-individual variability of clopidogrel has been frequently reported and has been regarded as “clopidogrel resistance” and HTPR [21]. Hyporesponse to clopidogrel with a high risk of coronary ischaemia and hyper response to clopidogrel with a high risk of bleeding results in an increase in cardiovascular events. Clinical factors including diabetes, poor absorption, and drug-drug interactions may be one aspect of HTPR [21]. Genetic factors are another aspect to consider. The polymorphism of dominant genes such as *CYP2C19, ABCB1*, *P2Y12*, and *T2238C* result in HTPR, especially in Chinese patients [22–26]. Furthermore, the gene-based individualised clopidogrel strategy demonstrated no benefit from GRAVITAS [27], ARCTIC [28], Trigger-PCI [29], and ANTARCTIC [30].

In all patients in the present study, during the 1-year follow-up, INR was rigorously monitored and was within the therapeutic range. Our results from the present study demonstrate that the TT-induced bleeding risk will fall within acceptable ranges if INR is rigorously monitored. Likewise, the ACTIVE-W trial recorded a significant benefit from anticoagulation combined with DAPT in AF patients. The ACTIVE-W study also demonstrated that patients benefitted from anticoagulation therapy, with INR values within the therapeutic range [31].

Another problem is the use of the drug-eluting stent (DES) for AF patients, which is the predominant implantation scaffold for coronary intervention. DES use is also inevitably associated with delayed neointimal recovery and an increased risk of late ST, resulting in prolonged DAPT for up to 1 year. However, in patients requiring oral anticoagulation, prolonged DAPT may increase major bleeding risk [14]. Therefore, the use of bare metal stents (BMS) was suggested by some studies for such patients [32, 33] and DES should be implanted only in situations such as long lesions, small vessels, and diabetes, in which the significant advantages of DES outweigh the disadvantages of BMS [34]. In our study, the scaffold used was exclusively DES. Individualised clopidogrel MD and close INR monitoring might overcome the disadvantages of DES in long-term antithrombotic therapy.

In the SPORTIF trial [35], the addition of aspirin to oral anticoagulants (INR 2.0–3.0) increased major bleeding risk without decreasing thromboembolism or AMI in AF patients. In addition, results from the WOEST trial [36] showed a significant increase in death and MI events in those who received vitamin K antagonist plus DAPT than in those treated with vitamin K antagonist plus clopidogrel. These results demonstrate that in patients whose bleeding risk surpasses stroke risk, combined warfarin and clopidogrel would be a reasonable treatment choice. In the present study, the average HAS-BLED score was 3.1, which indicated a relatively high-bleeding cohort. Consequently, we chose the close monitoring of warfarin and modified clopidogrel plus fixed aspirin for 3 months. The strategy was also in accordance with the consensus of antithrombotic therapy in AF patients with a combination of ACS and/or undergoing PCI [2].

The interaction of warfarin and clopidogrel was investigated in the Sibbing et al. study [37], in which they reported that both clopidogrel and phenprocoumon were metabolised synchronously through the hepatic cytochrome P450 system and there was an intra-drug interaction at this level. The result demonstrated that phenprocoumon notably attenuated the antiplatelet effect of clopidogrel. If this is true, the VASP-guided clopidogrel therapy compensated for the attenuation of warfarin on antiplatelet effects in the current study.

To date, only two studies have investigated the effect of prasugrel on TT strategy. In the Sarafoff et al. study [38], aspirin, prasugrel, and vitamin K antagonist were prescribed for 6 months. Compared to clopidogrel, prasugrel increased the bleeding risk in patients requiring TT. In the TRANSLATE-ACS study [39] of AMI patients treated with PCI, clopidogrel or prasugrel was administered in addition to aspirin and anticoagulant. The study concluded that among patients with TT, prasugrel administration was associated with a higher bleeding risk. Thus, prasugrel was not recommended in the consensus document.

Because of the high bleeding risk, a new oral anticoagulant therapy was evaluated. The RE-DUAL PCI study [40] compared TT with warfarin plus a P2Y12 antagonist (clopidogrel or ticagrelor) and aspirin (TT group) to dual therapy with dabigatran plus a P2Y12 antagonist (clopidogrel or ticagrelor) and no aspirin. The results demonstrated that among AF patients undergoing PCI, the bleeding risk was lower in patients who received the latter combination than in patients who received the former. Dual therapy was non-inferior to TT with respect to the risk of thromboembolism. In the PIONEER AF-PCI study [41], the combination of either low-dose rivaroxaban plus a P2Y12 antagonist or very-low-dose rivaroxaban plus DAPT significantly decreased the bleeding risk compared to standard therapy with a vitamin K antagonist plus DAPT for 1, 6, or 12 months. These two studies met the objective of decreasing bleeding risk; however, they exhibited no significant difference regarding thrombotic events. In addition, neither dabigatran nor rivaroxaban is used regularly in the real world, especially in China.

### Limitations

There are some limitations to the present study. First, the study was conducted in the Chinese population, which demonstrates more “clopidogrel resistance” than the European and North American populations, so the conclusion should be cautiously extended to others. Second, the sample of patients recruited in the present study was relatively small. Third, the present study did not evaluate the effects of the new antiplatelet ticagrelor or new anticoagulants such as dabigatran or rivaroxaban. In the future, we will focus on the antithrombotic effect of these antagonists.

## Conclusion

The present study verified that a modified clopidogrel maintenance strategy combined with aspirin and warfarin therapy decreased adverse cardiovascular events at the cost of increasing minor bleeding.

## Abbreviations

ADP: adenosine diphosphate
AF: atrial fibrillation
AMI: Acute myocardial infarction
DAPT: dual antiplatelet therapy
DES: drug-eluting stent
HTPR: high on-treatment platelet reaction
INR: international normalised ratio
MACCE: major adverse cardiovascular and cerebral events
MD: maintenance dose
MFI: mean fluorescence intensity
PCI: percutaneous coronary intervention
PGE1: prostaglandin E1
PRI: platelet reactivity index
ST: stent thrombosis
TIMI: thrombolysis in myocardial infarction
TT: triple therapy
TVR: target vessel revascularisation
VASP: vasodilator-stimulated phosphoprotein
